# Supplementary material for: Epidemiology of Status Epilepticus in Kazakhstan: A 10-Year Population-Based Study
Source: J Clin Med. 2025 Dec 17;14(24):8911. doi: 10.3390/jcm14248911 (PMC12733524; doi:10.3390/jcm14248911)
Supplement: Supplementary file 1 [file jcm-14-08911-s001.zip › jcm-4024632-supplementary.pdf]

Supplementary Materials

Supplementary Table S1. Covariates and their corresponding ICD-10 codes

**Covariate**	**ICD-10 Code**
Epilepsy	G40.0-G40.9
Cerebrovascular diseases	I60-I69
Central nervous system infection	A06.6, A17, A39, A80-A89, B00.3, B00.4, B01.0, B01.1, B02.0, B02.1, B05.0, B05.1, B06.0, B22.0, B26.1, B26.2, B37.5, B38.4, B43.1, B50.0, B58.2, B60.2, G00-G09, R29.1
Brain tumor	C71, D33, D43
Cancer	C00-C97

Supplementary Table S2. Total number of hospital admissions due to status epilepticus per patient from 2014 to 2023.

**Total admissions count**	**Alive**	**Deceased**	**Total**
1	10,019 (89.91 %)	1,124 (10.09 %)	11,143 (100.00 %)
2	1,601 (91.38 %)	151 (8.62 %)	1,752 (100.00 %)
3	500 (90.25 %)	54 (9.75 %)	554 (100.00 %)
4	204 (91.07 %)	20 (8.93 %)	224 (100.00 %)
5	106 (93.81 %)	7 (6.19 %)	113 (100.00 %)
6	74 (92.50 %)	6 (7.50 %)	80 (100.00 %)
7	39 (95.12 %)	2 (4.88 %)	41 (100.00 %)
8	29 (93.55 %)	2 (6.45 %)	31 (100.00 %)
9	21 (87.50 %)	3 (12.50 %)	24 (100.00 %)
10	11 (73.33 %)	4 (26.67 %)	15 (100.00 %)
11	6 (100.00 %)	0 (0.00 %)	6 (100.00 %)
12	5 (83.33 %)	1 (16.67 %)	6 (100.00 %)
13	2 (100.00 %)	0 (0.00 %)	2 (100.00 %)
14	3 (100.00 %)	0 (0.00 %)	3 (100.00 %)
15	6 (100.00 %)	0 (0.00 %)	6 (100.00 %)
16	1 (100.00 %)	0 (0.00 %)	1 (100.00 %)
17	1 (100.00 %)	0 (0.00 %)	1 (100.00 %)
18	1 (100.00 %)	0 (0.00 %)	1 (100.00 %)
19	0 (0.00 %)	1 (100.00 %)	1 (100.00 %)
21	1 (100.00 %)	0 (0.00 %)	1 (100.00 %)
22	0 (0.00 %)	1 (100.00 %)	1 (100.00 %)
24	1 (100.00 %)	0 (0.00 %)	1 (100.00 %)
25	3 (100.00 %)	0 (0.00 %)	3 (100.00 %)
**Total**	**12,634 (90.18 %)**	**1,376 (9.82 %)**	**14,010 (100.00 %)**

Supplementary Table S3. Age-standardized incidence and hospital admissions rates for status epilepticus in Kazakhstan from 2014 to 2023.

	**First episode (n=14,010)**		**Hospital Admissions (n=19,659)**	
**Year**	**ASIR (95% CI)**	**APC (95% CI)**	**ASIR* (95% CI)**	**APC (95% CI)**
2014	3.90 (3.61; 4.20)	0	4.15 (3.85; 4.46)	0
2015	6.61 (6.26; 6.98)	69.97 (54.67; 86.28)	7.92 (7.52; 8.32)	91.23 (74.51; 108.93)
2016	7.70 (7.30; 8.11)	16.52 (7.89; 25.55)	9.75 (9.30; 10.19)	23.18 (14.85; 31.86)
2017	7.62 (7.24; 8.01)	-1.02 (-7.89; 6.29)	10.15 (9.71; 10.61)	4.22 (-2.19; 11.02)
2018	7.49 (7.10; 7.88)	-1.55 (-8.38; 5.82)	10.40 (9.93; 10.88)	2.48 (-3.80; 9.01)
2019	6.94 (6.60; 7.31)	-7.26 (-13.70; -0.24)	9.95 (9.52; 10.40)	-4.29 (-10.25; 1.93)
2020	7.38 (7.01; 7.77)	6.40 (-1.07; 14.28)	10.95 (10.49; 11.41)	10.11 (3.53; 16.91)
2021	11.79 (11.32; 12.27)	59.78 (49.82; 70.43)	17.02 (16.45; 17.59)	55.54 (47.42; 64.08)
2022	8.37 (7.97; 8.77)	-29.02 (-33.36; -24.54)	12.69 (12.19; 13.19)	-25.43 (-29.15; -21.49)
2023	7.20 (6.83; 7.57)	-13.94 (-19.63; -7.84)	12.12 (11.64; 12.62)	-4.42 (-9.59; 1.11)

ASIR: age-standardized incidence (first episode) rate; ASIR*: age-standardized hospitalizations rate; APC: annual percent change; Incidence (first episode) AAPC during 2014-2023: 11.10% (-10.53 % to 32.73 %); Admissions AAPC during 2014-2023: 16.96% (-6.33 % to 40.26 %);

Supplementary Table S4. Difference in survival time and its ratio from multivariable analyses using restricted mean survival time method.

Covariate		30-day mortality	1-year mortality	2-year mortality	3-year mortality	4-year mortality	5-year mortality
**Age**							
Age 60 or older	AD (95% CI)	-1.07 (-1.36; -0.77), p <0.001	-39.21 (-45.06; -33.37), p <0.001	-108.16 (-121.49; -94.83), p <0.001	-197.44 (-219.03; -175.85), p <0.001	-298.84 (-329.26; -268.42), p <0.001	-408.73 (-448.46; -369.01), p <0.001
	Ratio (95% CI)	0.96 (0.95; 0.97), p <0.001	0.88 (0.87; 0.90), p <0.001	0.84 (0.82; 0.86), p <0.001	0.81 (0.79; 0.83), p <0.001	0.78 (0.76; 0.80), p <0.001	0.76 (0.73; 0.78), p <0.001
**Sex**							
Male sex	AD (95% CI)	-0.02 (-0.13; 0.08), p = 0.65	-1.01 (-3.02; 0.99), p = 0.32	-6.88 (-11.42; -2.34), p = 0.001	-16.31 (-23.76; -8.84), p <0.001	-27.16 (-37.82; -16.50), p <0.001	-40.37 (-54.55; -26.19), p <0.001
	Ratio (95% CI)	0.99 (0.99; 1.00) p = 0.65	0.99 (0.99; 1.00), p = 0.32	0.99 (0.98; 0.99), p = 0.001	0.98 (0.97; 0.99), p <0.001	0.98 (0.97; 0.98), p <0.001	0.97 (0.96; 0.98), p <0.001
**Epilepsy**							
Coexisting epilepsy	AD (95% CI)	0.28 (0.17; 0.39), p <0.001	6.82 (4.71; 8.93), p <0.001	13.86 (8.92; 18.81), p <0.001	19.20 (10.77; 27.63), p <0.001	17.92 (5.50; 30.34), p <0.001	8.76 (-8.15; 25.68), p =0.31
	Ratio (95% CI)	1.01 (1.00; 1.01), p <0.001	1.02 (1.01; 1.02), p <0.001	1.02 (1.01; 1.02), p <0.001	1.01 (1.01; 1.02), p <0.001	1.01 (1.00; 1.02), p <0.001	1.01 (0.99; 1.02), p = 0.27
**Cerebrovascular diseases**							
History of CVD	AD (95% CI)	-0.06 (-0.30; 0.18), p = 0.61	-2.43 (-7.31; 2.44), p = 0.32	-10.25 (-21.72; 1.22), p =0.08	-22.43 (-42.18; -2.69), p = 0.03	-43.89 (-73.05; -14.72), p <0.001	-63.49 (-104.07; -22.91), p = 0.002

	Ratio (95% CI)	0.99 (0.98; 1.01), p = 0.61	0.99 (0.97; 1.01), p =0.33	0.98 (0.96; 1.00), p = 0.08	0.97 (0.95; 0.99), p = 0.03	0.96 (0.94; 0.98), p <0.001	0.96 (0.93; 0.98), p = 0.002
**Central nervous system infection**							
History of CNS infection	AD (95% CI)	-0.06 (-0.43; 0.31), p = 0.75	-7.14 (-15.35; 1.06), p = 0.08	-23.99 (-43.26; -4.73), p = 0.01	-41.69 (-73.26; -10.12), p = 0.01	-57.75 (-102.69; -12.82), p = 0.01	-70.42 (-129.73; -11.11), p = 0.02
	Ratio (95% CI)	0.99 (0.98; 1.01), p = 0.75	0.98 (0.95; 1.00), p = 0.09	0.96 (0.93; 0.99), p = 0.01	0.96 (0.93; 0.99), p = 0.01	0.95 (0.92; 0.99), p = 0.01	0.95 (0.92; 0.99), p = 0.02
**Brain tumor**							
History of brain tumor	AD (95% CI)	-0.29 (-0.83; 0.24), p = 0.28	-34.37 (-48.08; -20.66), p <0.001	-95.27 (-127.13; -63.40), p <0.001	-168.34 (-220.72; -115.96), p <0.001	-251.26 (-326.19; -176.33), p <0.001	-328.15 (-430.17; -226.13), p <0.001
	Ratio (95% CI)	0.99 (0.97; 1.01), p = 0.28	0.90 (0.86; 0.94), p <0.001	0.86 (0.81; 0.90), p <0.001	0.83 (0.78; 0.88), p <0.001	0.81 (0.75; 0.86), p <0.001	0.79 (0.73; 0.86), p <0.001
**Cancer**							
History of cancer	AD (95% CI)	-1.16 (-2.11 - 0.21), p = 0.01	-43.20 (-62.90; -23.50), p < 0.001	-119.38 (-163.36; -75.41), p <0.001	-219.62 (-289.28; -149.95), p <0.001	-326.21 (-423.15; -229.27), p <0.001	-441.69 (-567.04; -316.34), p <0.001
	Ratio (95% CI)	0.96 (0.92; 0.99), p = 0.01	0.87 (0.81; 0.93), p <0.001	0.81 (0.75; 0.88), p <0.001	0.77 90.70; 0.84), p <0.001	0.73 (0.66; 0.81), p <0.001	0.71 (0.63; 0.79), p <0.001

CAPTION. AD: adjusted difference in days; CI: confidence interval; CVD: cerebrovascular diseases; CNS: Central nervous system; Ratio: stands for the ratio of survival time in two contrasted groups; All estimates are adjusted for age, sex, epilepsy, CVD, CNS infections, brain tumor, and cancer. Reference groups are: age less than 60, female sex, and absence of epilepsy, CVD, CNS infection, brain tumor, and cancer;

Supplementary Figure S1. Time-specific hazard ratio from Cox regression models for epilepsy and cerebrovascular diseases covariates.

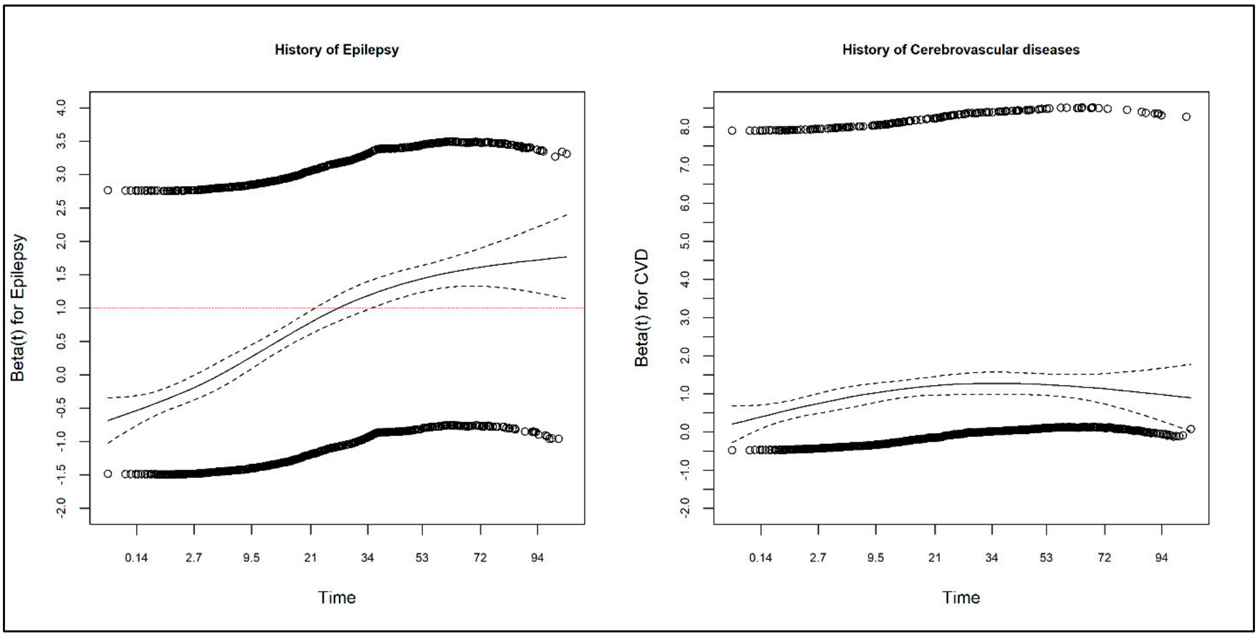

CAPTION: in both cases, the HR was lower in those who had epilepsy diagnosis or a history of cerebrovascular diseases. However, as time (in months) passed, the HR gradually increased, suggesting violation of proportional hazards assumption. In addition, Schoenfeld test revealed $p < 0.001$ for both covariates.
